# MYC is a critical target of FBXW7

**DOI:** 10.18632/oncotarget.3203

**Published:** 2014-12-26

**Authors:** Mai Sato, Ruth Rodriguez-Barrueco, Jiyang Yu, Catherine Do, Jose M. Silva, Jean Gautier

**Affiliations:** ^1^ Department of Pathology and Cell Biology, Columbia University, New York, USA; ^2^ Institute for Cancer Genetics, Columbia University, New York, USA; ^3^ Department of Biomedical Informatics, Columbia University, New York, USA; ^4^ Department of Genetics and Development, Columbia University, New York, USA; ^5^ Department of Pathology, Mount Sinai School of Medicine, New York, USA

**Keywords:** MYC, FBXW7, synthetic lethality, CDC45, MCF10A

## Abstract

MYC deregulation is a driver of many human cancers. Altering the balance of MYC protein levels at the level of transcription, protein stability, or turnover is sufficient to transform cells to a tumorigenic phenotype. While direct targeting of MYC is difficult, specific genetic vulnerabilities of MYC-deregulated cells could be exploited to selectively inhibit their growth. Using a genome-wide shRNA screen, we identified 78 candidate genes, which are required for survival of human mammary epithelial cells with elevated MYC levels. Among the candidates, we validated and characterized FBXW7, a component of the SCF-like ubiquitin ligase complex that targets MYC for proteasomal degradation. Down-regulation of FBXW7 leads to synergistic accumulation of cellular and active chromatin-bound MYC, while protein levels of other FBXW7 targets appear unaffected. Over a four-week time course, continuous FBXW7 down-regulation and MYC activation together cause an accumulation of cells in S-phase and G2/M-phase of the cell cycle. Under these conditions, we also observe elevated chromatin-bound levels of CDC45, suggesting increased DNA replication stress. Consistent with these results, FBXW7 down-regulation alone decreases the survival of T47D breast cancer cells. These results establish that FBXW7 down-regulation is synthetic lethal with MYC, and that MYC is a critical target of FBXW7 in breast epithelial cells.

## INTRODUCTION

Genetic and epigenetic events altering the expression, the stability, or the activity of oncogenes and tumor suppressor genes participate in the initiation and maintenance of human cancers [1]. Among the genes which expression is modified in cancer, only a relatively small subset is recurrently altered and contributes to the tumorigenic phenotype [2]. Identification of such “cancer driver genes” has facilitated the development of targeted treatment options in the recent years, yet there are still pharmacological limitations that hinder direct targeting of some major cancer drivers including MYC [3].

The MYC proto-oncogene is deregulated in over half of human cancers including hematopoietic cancers, sarcomas, and carcinomas [4]. MYC is essential for normal cell growth. In particular, MYC is required for cell proliferation and is involved in differentiation, metabolism, and apoptosis, most notably through its function as a bHLH-LZ transcriptional activator and repressor [5]. In addition, MYC has direct, transcription-independent functions in DNA replication [6] and protein synthesis [7], further supporting its major role in cellular homeostasis. Therefore, cellular MYC protein levels are tightly controlled through regulated expression [8], protein stability [9], and degradation [10]. Indeed, alterations in MYC abundance are a critical component of MYC-dependent tumorigenesis [11].

At least 4 different ubiquitin ligase complexes can target MYC for proteasomal degradation [10], including the SCF-like ligase complex containing FBXW7 [12, 13]. FBXW7 (or Fbw7, Sel-10, hCdc4, hAgo) is an F-box protein that confers substrate specificity for this complex [14]. MYC is a documented target of FBXW7. However, FBXW7 also targets several additional oncoproteins and master regulator molecules including CyclinE, NOTCH1, mTOR, SREBP, and c-JUN, among others [14] and the respective contributions of FBXW7-dependent regulation of these proteins towards abnormal cell growth is not known. Moreover, although the role of FBXW7 as a tumor suppressor is well documented in hematopoietic tumors in which mutations in FBXW7 are unequivocally linked to tumorigenesis [15], this is not as clear for other cancer types [16, 17]. For example, it has been reported that the inactivation of FBXW7 can be associated with favorable prognosis in a subset of breast cancers [18], suggesting that the physiological role of FBXW7 and consequences of its loss may be dependent on cell types and contexts.

Exploring synthetic lethality has provided many critical insights into the biology of oncogenes [19]. In addition, identifying genes that are essential to cope with activated oncogenes might provide alternatives to direct targeting for cancer therapy [20]. In the case of MYC, several genes which loss is synthetic lethal with aberrant MYC expression have been identified. These include DR5 in the death receptor pathway [21], the WRN helicase [22, 23], the AuroraA/B [24] and CDK [25, 26] cell-cycle kinases, and Chk1/2 [27, 28]. More recently, genome-wide approaches have helped to identify the SAE1/2-dependent SUMOylation pathway [29] and CSNK1E kinase [30] as potential synthetic lethal candidates as well. Intriguingly, studies in different model systems appear to yield unique results, possibly due to MYC's cell-context specific roles and the differing modes of deregulation.

In the present study, we conducted a genome-wide screen to identify genes that are required to survive MYC overexpression in MCF10A breast epithelial cells conditionally expressing a MYCER allele. We document these synthetic lethal interactions and identify 78 potential synthetic lethal targets. Among these, we have validated the F-box protein FBXW7 as a synthetic lethal candidate in MCF10A-MYCER cells overexpressing MYC. We show that shRNA-mediated knockdown of FBXW7 confers increased lethality to non-transformed MCF10A cells with activated MYCER and to T47D breast cancer cells. In MCF10A, active chromatin-bound MYCER is stabilized in cells with down-regulated FBXW7, while protein stability of other FBXW7 targets is not affected. We also find that knockdown of FBXW7 upon MYCER activation results in accumulation of cells in S/G2 phase with increased chromatin-bound CDC45. These results suggest that increased cellular MYCER levels as a result of FBXW7 loss yields sufficient cellular stress to confer selective lethality to those cells with deregulated MYC.

## RESULTS

### MCF10A-MYCER cells: a model for MYC deregulation

First, we sought to generate a cellular model for MYC conditional expression that recapitulates MYC deregulation in human cancer cells. To this goal, we stably integrated an inducible MYCER transgene [31] into MCF10A, a non-transformed breast epithelial cell line, via retroviral transduction. MYCER consists of a full-length MYC cDNA fused to the hormone-binding domain of the murine estrogen receptor. This ER fragment does not respond to estrogen [31]. Upon treatment with the synthetic ligand 4-hydroxytamoxifen (4OHT), MYCER protein is rapidly transported to the nucleus, rendering it active.

**Figure 1:**
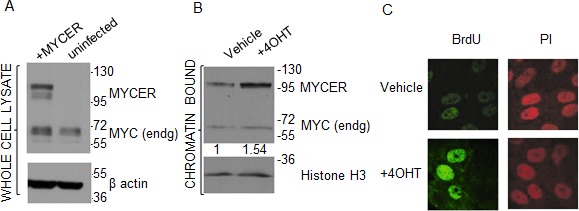
MCF10A-MYCER cells: a model for MYC deregulation (A) MCF10A-MYCER cells express MYCER protein. Whole cell lysates from uninfected or MYCER-infected MCF10A cells were analyzed by SDS-PAGE. The positions of endogenous MYC and MYCER are indicated. (B) MYCER protein is activated in response to 4OHT treatment. Nuclear translocation of MYCER was analyzed by cell fractionation followed by SDS-PAGE of the chromatin-bound fraction from MCF10A-MYCER cells treated with vehicle or 200nM 4OHT for 48 hr. MYCER increase was normalized to histone H3 levels for quantification. (C) MYCER activation induces accelerated entry into S phase. MCF10A-MYCER cells were arrested at G1/S with double thymidine. Cells were treated with vehicle (ethanol) or 4OHT, then pulse labeled with BrdU 1 hr after release into S phase. BrdU incorporation was assessed using indirect immunofluorescence. PI=propidium iodide was used to stain genomic DNA.

Following retroviral transduction, we selected a clone expressing MYCER (Figure 1A) that fulfilled two criteria: 1) normal levels of endogenous MYC expression were retained, and 2) MYCER was expressed at levels 2-3 fold higher than endogenous MYC, a level of MYC expression that is appropriate for modeling MYC deregulation in human cancers without MYC amplification [32] (Figure 1A). Following 4OHT treatment, this clone showed a 1.54 fold increase in active, chromatin-bound MYCER, demonstrating the anticipated 4OHT-dependent nuclear import and activation of MYCER (Figure 1B). Next, we tested the ability of the MYCER transgene to activate transcription. MCF10A-MYCER cells were transfected with a luciferase reporter construct harboring tandem E-box sequences, the canonical MYC DNA binding site (Supplementary Figure 1). Upon 4OHT treatment, we observed a 2.3 fold increase in reporter activity (p<0.0001), consistent with the increase in chromatin-bound MYCER (Supplementary Figure 1). Finally, we tested the ability of the transgene to accelerate entry into S-phase, another hallmark of MYC deregulation. Cells were synchronized at the G1/S transition by double thymidine block and labeled with BrdU following induction of MYCER. S-phase entry was then monitored by the appearance of BrdU foci assessed by indirect immunofluorescence. As anticipated, MYCER induction triggered marked acceleration of S-phase entry (Figure 1C and Supplementary Figure 2). These results establish the validity of MCF10A-MYCER to model a near physiological degree of MYC deregulation.

### Screen for synthetic lethal candidates with MYCER activation

**Figure 2:**
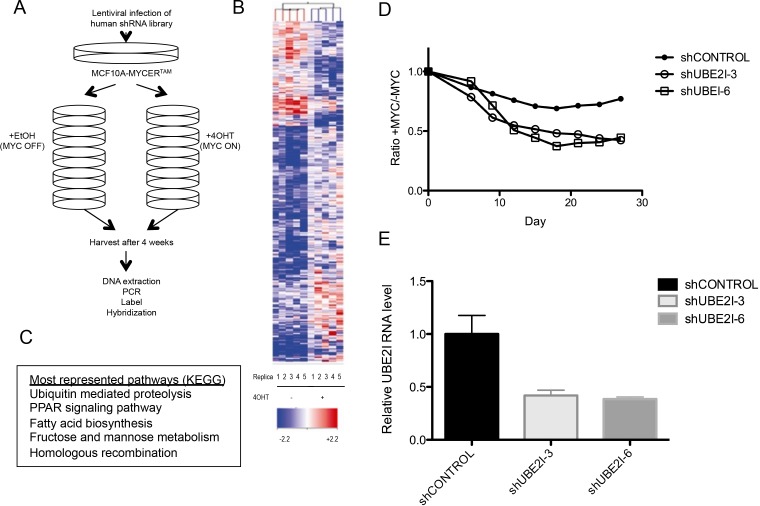
Genome-wide screen for synthetic lethal candidates with MYCER activation (A) Schematic diagram of screen design. See text for details. (B) Identification of synthetic lethal shRNA with MYCER. Heat map shows the relative enrichment or depletion of each shRNA in all experimental replicates (5 for MYC OFF and 5 for MYC ON). The spectrum between red and blue denote the relative representation of each shRNA barcode in the sample compared to the reference (library at T=0). (C) Five biological pathways most required for the survival of MCF10A-MYCER. Pathway analysis of the top synthetic lethal candidates (Log2 FC<-1) shows enrichment of genes in five biological pathways. Pathways are listed in order of number of genes appearing from the dataset. (D) UBE2I knockdown is synthetic lethal with MYCER activation. MCF10A-MYCER cells stably expressing two independent shRNA clones targeting UBE2I (shUBE2I-3 and shUBE2I-6) were subjected to competitive survival assays. Fluorescence-based competition assay data are plotted as ratios of %GFP of MYC ON cells to MYC OFF cells at the given time points for cell lines stably expressing the indicated shRNA. Graph represents the average of three replicates (p<0.0001 between shCONTROL and shUBE2I-3, p<0.0001 between shCONTROL and shUBE2I-6). (E) shRNA-mediated stable knockdown of UBE2I. MCF10A-MYCER cells stably expressing shRNA clones targeting UBE2I (UBE2I-3 and UBE2I-6) were subjected to quantitative RT-PCR. Error bars represent the SEM of 3 independent experiments.

Next, we performed a genome-wide screen to identify genes which loss is synthetic lethal with aberrant MYC expression utilizing the GIPZ Lentiviral Human shRNA-mir Library (Thermo Scientific Open Biosystems, Waltham, MA, USA). The pooled shRNA plasmid library includes 58,493 shRNA constructs and targets 18,661 known human genes (2-3 shRNAs per gene covering 75% of entire genome) as described in Rodriguez-Barrueco et al [33]. We designed the screen based on negative selection, i.e. we monitored loss of shRNAs to identify the corresponding genes that render cells sensitive to MYCER activation by 4OHT when lost (Figure 2A).

The screen was performed in 5 independent replicates, which were utilized to generate p-values for screening of statistically significant high-confidence candidates. Cells were initially transduced with the pooled shRNA library at 30% infection efficiency to achieve, on average, single-copy shRNA integration per cell. 200 million cells were infected in order to obtain at least 1000 cells with each shRNA from the library. Infected MCF10A-MYCER cells were first allowed to grow in the absence of MYCER induction for two generations to eliminate most shRNAs targeting essential genes. Then, the remaining cells were propagated in either the presence or absence of 4OHT for four weeks, maintaining a constant total cell number of at least 70 million per sample to preserve the representation of at least 1000 cells per shRNA construct. The surviving populations were harvested after four weeks, and genomic DNA was isolated (Figure 2A). The stably integrated shRNA constructs were then amplified by large scale PCR, labeled with fluorescent nucleotides, and hybridized to custom microarrays harboring complementary oligonucleotide probes to the unique 60-nucleotide barcodes present on each individual shRNA.

The microarray analysis was performed as described in Yu et al [34] (Figure 2B). We identified 78 candidate genes for which the corresponding shRNAs had a Log2FC (fold change) value of −1 or less, thus, depleted 2-fold or more in the MYC ON population compared to MYC OFF with a p-value of p<0.05 across five replicates (Supplementary Table 1). Functional annotation clustering using the KEGG pathways in the DAVID Bioinformatics Database revealed that the most represented biological pathways within the synthetic lethal candidates included ubiquitin mediated proteolysis, PPAR signaling pathway, fatty acid biosynthesis, fructose and mannose metabolism, and homologous recombination (Figure 2C). Within the proteolysis pathway and among the top hits we identified UBE2I, the E2 conjugating enzyme cooperating with the SAE1/2 E3 ligase in the SUMOylation pathway. This is in agreement with previous reports in which SAE1/2 and UBE2I were identified in similar MYC-synthetic lethal (MYC-SL) screens [29, 30]. Using a set of independent shRNAs, we validated that loss of UBE2I is indeed synthetic lethal with MYCER activation in MCF10A-MYCER cells (p<0.0001), providing a proof of principle for our screen (Figures 2D and 2E).

Notably, three major MYC-SL screens have been published to date [29, 30, 35]. When comparing our dataset to these screens, we observed that FBXW7 was one of four MYC-SL hits that overlapped between our screen and Kessler's screen [29]. Additionally, the GSK3-β kinase, which is required for MYC degradation by FBXW7, was found as one of the eleven top MYC-SL kinases from Liu's screen [12, 35]. Moreover, the MYC ubiquitination and SUMOylation network was determined as one of three major functional MYC-SL hubs, in a comprehensive meta-analysis of the three published MYC-SL datasets [36]. These results suggest that FBXW7-dependent MYC degradation may be a significant MYC-SL pathway. Therefore, we selected FBXW7 for further analysis, which ranked 58^th^ in our high-confidence MYC-SL list with Log2FC=-1.1469 (p<0.02, Supplementary Table 1).

### Loss of FBXW7 together with MYCER activation is lethal

**Figure 3:**
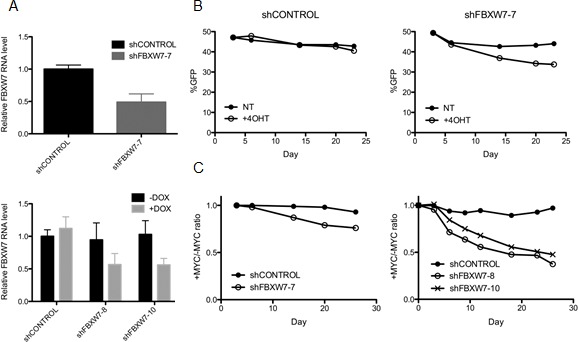
Loss of FBXW7 combined with MYCER activation is lethal (A) Knockdown of FBXW7 in MCF10A-MYCER cells. FBXW7 expression was inhibited in MCF10A-MYCER cells using lentiviral transduction of shRNA. Constitutive (FBXW7-7) (top panel) and inducible (FBXW7-8 and -10) (bottom panel) knockdown levels were analyzed by quantitative RT-PCR. Error bars represent the SEM of 3 independent experiments. (B) FBXW7 knockdown compromises the viability of MYC ON cells. Fluorescence-based competition assay over 23 days was performed as described in the text. The GFP-expressing population was measured at the indicated time points for untreated (NT) and 4OHT-treated cells. Cells were treated with vehicle or 4OHT every 72 hr for the duration of the experiments. Graphs represent the average of three replicates (NT vs 4OHT not significant for shCONTROL; p=0.0006 for shFBXW7-7). (C) Three independent clones of FBXW7 shRNA decrease the viability of 4OHT-induced MCF10A-MYCER cells. Fluorescence-based competition assay data are plotted as ratios of %GFP of MYC ON to MYC OFF at the given time points. Both constitutive (left panel) and inducible (right panel) FBXW7 knockdown clones show synthetic lethal effect with MYC activation. Graphs represent the average of three replicates (p=0.0003 between shCONTROL and shFBXW7-7 in left panel; p<0.0001 for both shFBXW7-8 and shFBXW-10 in right panel).

We elected to further validate FBXW7, the substrate recognition subunit of the SCF-like E3 ubiquitin ligase complex that regulates degradation of MYC and other oncoproteins [14]. First, we wanted to confirm that loss of FBXW7 is lethal when combined with MYCER activation. We generated cell lines stably expressing either constitutively expressed or doxycycline-inducible shRNA clones targeting different regions of FBXW7 to experimentally manipulate its abundance. Following lentiviral infection of stable shRNA constructs and antibiotic selection, FBXW7 knockdown levels were assessed by quantitative RT-PCR. Both constitutive and doxycycline-inducible shRNA clones yielded an average of 45% knockdown of FBXW7 mRNA levels (Figure 3A). We were unable to achieve further knockdown of FBXW7 in MCF10A-MYCER cells, suggesting a threshold level of FBXW7 is required for MCF10A-MYCER viability, even when the MYC transgene is off.

We utilized a fluorescence-based cell competition assay to compare the viability of FBXW7 knockdown cells in the absence or presence of 4OHT over a period of approximately four weeks, to recapitulate the experimental conditions of the screen and physiological modest deregulation of MYC. Since all shRNA constructs co-express GFP or RFP, cells expressing shRNA are readily detectable by fluorescence. A mixed population of 50% MCF10A-MYCER cells without shRNA and 50% MCF10A-MYCER cells infected with shFBXW7 or shCONTROL were plated in MYC OFF or MYC ON conditions and continuously treated with vehicle or 4OHT at every passage. Cells were harvested at the indicated time points during the competition assay (Figures 3B and 3C), and subjected to live cell flow cytometry to measure the percentage of fluorescent surviving cells (Supplementary Figure 3).

Both constitutive and inducible FBXW7 knockdown yielded a significant decrease in viability of cells treated with 4OHT expressing MYCER. Survival of cells infected with control shRNA was not significantly affected by MYCER induction over a period of 23 days, whereas cells with FBXW7 knockdown showed 11% less GFP signal in the presence of 4OHT by Day 23 (p=0.0006) (Figure 3B). When plotted as the MYC ON/MYC OFF ratio of surviving cells, we determined that the constitutive FBXW7 knockdown caused a 21% decrease in viability (p=0.0003), and the inducible knockdown clones resulted in an average of 53% decrease in viability compared to control cells (p<0.0001) (Figure 3C). The inducible clones likely yield a more robust reaction due to continuous induction of shRNA by 1μg/ml doxycycline addition at every passage.

Of note, all the shRNAs used in this secondary screen target different sequences within the FBXW7 mRNA from the shRNAs included the library, confirming that the observed phenotype is due to FBXW7 down-regulation. Taken together, these results validate the initial finding from our screen and establish that FBXW7 knockdown is synthetic lethal with MYCER activation.

### T47D breast tumor-derived cells are selectively sensitive to FBXW7 knockdown

**Figure 4:**
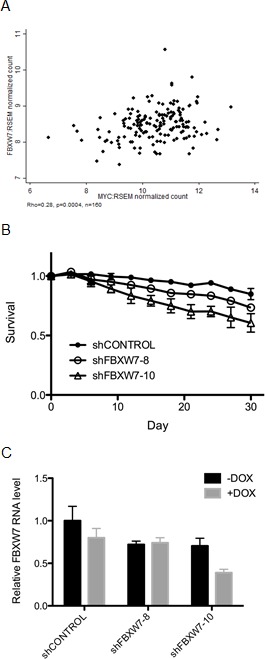
T47D breast cancer-derived cells are selectively sensitive to FBXW7 knockdown (A) FBXW7 and MYC expression levels positively correlate in Luminal A-type breast cancers. RNAseq (IlluminaHiSeq) data from TCGA breast invasive carcinoma dataset (TCGA_BRCA_exp_HiSeqV2) were clustered using a “cluster of clusters” approach. Expression of MYC and FBXW7 in LUMINAL A subtypes were estimated in RSEM normalized count and Pearson correlation coefficient (rho) was calculated using STATA. (B) FBXW7 knockdown decreases the viability of T47D cells. Fluorescence-based competition assay data are plotted as ratios of MYC ON to MYC OFF at the given time points. All cells were continuously induced with 1μg/ml doxycycline at every passage during the assay. Error bars represent the SEM of 3 replicates (p=0.0076 between shCONTROL and shFBXW7-8; p=0.01 between shCONTROL and shFBXW7-10). (C) Knockdown of FBXW7 in T47D cells. FBXW7 expression was down-regulated in T47D cells using lentiviral transduction of shRNA. shRNA expression was induced by 1μg/ml doxycycline and FBXW7 mRNA levels were assessed by RT-PCR. Error bars represent the SEM of 3 replicates.

Since we confirmed the synthetic lethal effect of FBXW7 knockdown with MYCER activation in MCF10A-MYCER cells, next we elected to examine the correlation between expression of FBXW7 and MYC in various breast tumor subtypes. Analysis of all breast tumors from The Cancer Genome Atlas (TCGA) database did not show any significant correlation between FBXW7 and MYC expression (data not shown). Intriguingly, however, we observed a modest, yet highly significant positive correlation between FBXW7 and MYC expression, exclusively in the Luminal A-subtype of breast cancers (rho=0.28, p=0.0004) (Figure 4A). Luminal A breast cancers are the most prevalent subtype, accounting for approximately 40% of breast cancer cases [37]. Since this subtype is not normally associated with MYC overexpression, it is possible that Luminal A breast cancers retain sensitivity to MYC protein abundance, unlike MYC-independent breast cancer subtypes.

To expand our observation to another cell type and test if Luminal A-type cells are hypersensitive to FBXW7 knockdown, we achieved stable integration of doxycycline-inducible FBXW7 shRNA in the T47D breast cancer-derived cell line, which harbors a Luminal A-like signature (ER/PR-positive, Her2-negative, overexpresses luminal-specific cytokeratins 8 and 18). Continuous knockdown of FBXW7 over 30 days in T47D cells caused up to 41% decrease in viability compared to control (p=0.0076 and p=0.01 for shFBXW7-8 and shFBXW7-10, respectively, compared to shCONTROL) (Figures 4B and 4C), consistent with our results in MCF10A-MYCER cells. We conclude from these observations that our initial findings from the screen using MCF10A-MYCER cells can be extended to the T47D breast cancer cell line in which MYC expression was not experimentally altered and independent of ER activation.

### FBXW7 knockdown specifically leads to stabilization of active MYCER

**Figure 5:**
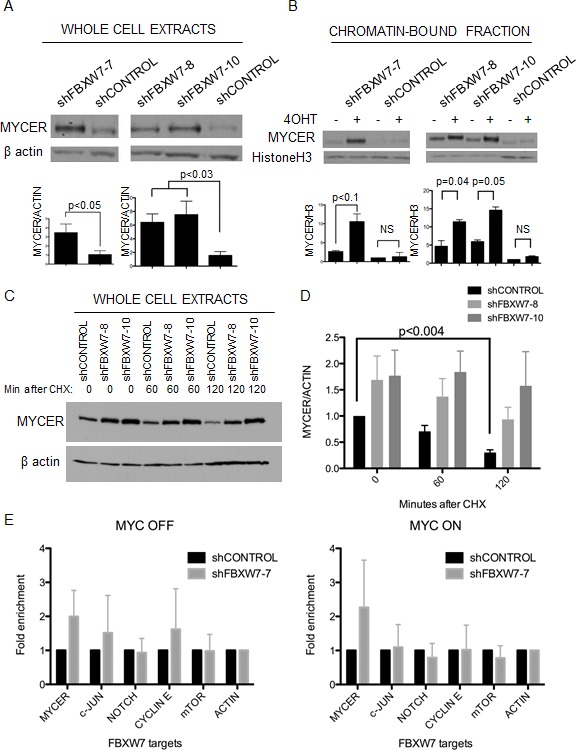
Loss of FBXW7 with MYCER activation results in specific stabilization of active MYCER protein (A) MYCER protein stability is enhanced in FBXW7 knockdown cells. Whole cell extracts were prepared from MCF10A-MYCER cells with FBXW7 knockdown. Inducible clones were activated with 1μg/ml doxycycline. Quantified values below each lane are normalized to β actin and averaged for 3 experiments. Error bars represent the SEM. (B) Active MYCER is stabilized on chromatin in FBXW7 knockdown cells following 4OHT induction. Cells treated with vehicle or 4OHT were fractionated, and fraction P3 (chromatin-bound) was analyzed on SDS-PAGE. Quantifications are normalized to histone H3 and values are averages of 3 experiments. Error bars represent the SEM. (C) MYCER protein is stabilized upon FBXW7 knockdown. Indicated cell lines were treated with 200nM 4OHT and 1μg/ml doxycycline for 24 h, then treated with 50μg/ml cycloheximide and harvested after 60 and 120 min. Whole cell lysates were run on SDS-PAGE. (D) Western blots from three independent experiments represented in (C) were normalized to β actin and quantified. Error bars represent the SEM. (E) FBXW7 knockdown with MYCER activation results in specific stabilization of MYCER. Whole cell extracts were prepared from control or FBXW7 knockdown cells and analyzed on SDS-PAGE followed by Western blot for FBXW7 targets (MYCER, c-Jun, Notch1, Cyclin E, mTOR). Quantifications show the fold enrichment of protein levels in control or FBXW7 knockdown cells normalized to β actin. Results are averages of 3 independent experiments and error bars represent the SEM.

To explore the mechanism by which FBXW7 down-regulation leads to lethality in MYC expressing cells, we first sought to assess whether down-regulation of FBXW7 resulted in the stabilization of MYCER. To this end, we examined the cellular and chromatin-bound levels of MYCER upon FBXW7 knockdown. Total cellular levels of MYCER were significantly increased upon down-regulation of FBXW7 with either constitutive or inducible FBXW7 shRNAs (3.5 fold and 6.2 fold, respectively, p<0.05) (Figure 5A). Moreover, the levels of active, chromatin-bound MYCER detected upon cell fractionation [38] were synergistically increased upon FBXW7 knockdown and MYCER activation (Figure 5B). Chromatin-bound MYCER increased 2-5 fold upon FBXW7 down-regulation and further increased up to 10-13.5 fold when MYCER was induced by 4OHT (p<0.1) (Figure 5B). To confirm if turnover of MYCER is affected by FBXW7 knockdown, we assessed total MYCER protein levels following cycloheximide treatment to prevent *de novo* MYCER synthesis in control or FBXW7 knockdown conditions. We observed increased stability of MYCER upon FBXW7 down-regulation, indicative of reduced protein turnover (Figure 5C and 5D). These results suggest that FBXW7 knockdown significantly stabilizes deregulated MYCER, resulting in elevated levels of active chromatin-bound MYCER.

As previously noted, FBXW7 controls the proteasome-dependent degradation of several cellular oncogenes which could also be stabilized upon FBXW7 knockdown and account for the observed phenotype [14]. Therefore, we examined the stability of other targets of FBXW7 by Western blot: c-Jun, NOTCH1, CyclinE, and mTOR, upon FBXW7 knockdown (Supplementary Figure 4). We find stabilization of MYCER, c-Jun, and CyclinE upon FBXW7 down-regulation in the absence of MYCER activation (Figure 5E, left). Notably, upon MYCER activation by 4OHT, only MYCER is stabilized when compared to the other FBXW7 targets examined (Figure 5E, right). Similar results were obtained using the inducible FBXW7 knockdown alleles (Supplementary Figure 5). These data suggest that FBXW7 knockdown in the context of MYCER activation leads predominantly to stabilization of MYCER. Our results point to the major role of FBXW7-mediated MYCER degradation for survival of cells with deregulated MYC.

### MYCER stabilization results in accumulation of chromatin-bound CDC45 and cells in S/G2 phase

**Figure 6:**
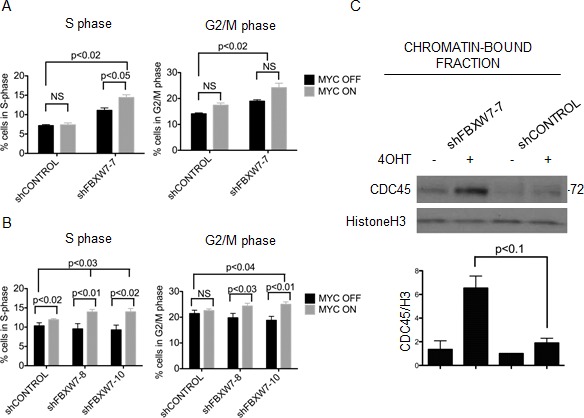
MYCER stabilization results in accumulation of cells in S/G2 phase and chromatin-bound CDC45 (A) Constitutive knockdown of FBXW7 with MYCER activation results in additive accumulation of cells in S and G2/M phase. MCF10A-MYCER cells with control or FBXW7 knockdown were cultured for 7 days in the absence or presence of 4OHT (MYC OFF or MYC ON). Cells were fixed and stained with propidium iodide for cell cycle analysis. Results are averages of 3 independent experiments and error bars represent the SEM. (B) Inducible knockdown of FBXW7 with MYCER activation results in accumulation of cells in S and G2/M phase. Control or FBXW7 knockdown cells were cultured for seven days with 1μg/ml doxycycline and vehicle or 4OHT every 72 hr. Results are averages of 3 independent experiments and error bars represent the SEM. (C) Loss of FBXW7 with MYCER activation causes accumulation of CDC45 on chromatin. MCF10A-MYCER cells with control or FBXW7 knockdown were cultured for 7 days with or without 4OHT and fractionated. Fraction P3 was analyzed on SDS-PAGE. Shown is a representative image of CDC45 blot, and graph shows the average of 3 independent experiments each normalized to histone H3. Error bars represent the SEM.

MYC deregulation triggers DNA damage and genomic instability [6, 39-41]. Therefore, we assessed the consequence of down-regulating FBXW7 together with MYCER activation on DNA damage and apoptosis. We monitored checkpoint activation (phosphorylation of Chk1 by ATR or Chk2 by ATM) and formation of DNA double strand breaks (phosphorylated H2AX), but could not detect a significant synergistic increase in these markers at any timepoint during the four week course of the experiments (Supplementary Figure 6A). Next, we probed for the apoptosis markers cleaved caspase-3, cleaved PARP, and PUMA, but did not detect significant changes by Western blotting (Supplementary Figure 6B). We then examined changes in cell cycle distribution. After four weeks of treatment with 4OHT, we found that FBXW7 knockdown cells in which MYC was deregulated showed synergistic accumulation of cells in S phase and G2/M phase (p<0.05) (Figure 6A, 6B, and Supplementary Figure 7). These data suggest that aberrant expression of MYC resulted in slower S-phase progression and/or DNA replication stress, and these phenotypes are exacerbated with FBXW7 knockdown.

We have recently demonstrated that MYC-dependent DNA replication stress is directly mediated by CDC45, a component of the replicative DNA helicase that marks active origins of replication. We documented the distribution of chromosomal origins of DNA replication and found that MYC or CDC45 expression both altered their patterns in *Xenopus* extracts and in B cells [42]. To assess if the cell cycle phenotype we observed could be attributed to aberrant S-phase progression as a result of accumulation of active MYCER on chromatin, we monitored the levels of chromatin-bound CDC45 in FBXW7 knockdown cells. We find that FBXW7 knockdown in the context of MYCER activation by 4OHT results in a synergistic increase in the levels of chromatin-bound CDC45 (6.7 fold increase, p<0.1), suggesting increased origin firing in the cells (Figure 6C). Our data suggest that FBXW7 knockdown cells with activated MYCER experience aberrant levels of origin firing during DNA replication, a sign of replication stress, which may contribute to the synthetic lethal phenotype.

## DISCUSSION

Genome-wide screening utilizing RNAi has become a powerful tool to assess genetic interactions in human cells. Three screens specifically designed to identify genes required for cell survival upon MYC deregulation have been previously published [29, 30, 35]. Kessler et al. expressed MYCER in HMEC cells and performed a pooled retroviral shRNA screen [29], Toyoshima et al. used isogenic HFF cells with or without a MYC transgene and performed an siRNA screen [30], and Liu et al. performed a screen using siRNA against the human kinome in U2OS cells [35]. Intriguingly, when we compare the 78 candidates from our screen to these datasets, less than 5% of genes overlap in pairwise comparisons. Between ours and Kessler's screen, four MYC-SL candidate genes overlap: FBXW7, LAMA2, PRMT8, and ZNF614 [29] (Supplementary Table 1). With Toyoshima's screen [30], we observed one overlap, UBE2I, which we (Figure 2) and Kessler et al. have validated [29, 30] (Figures 2D and 2E). With Liu's screen, we did not observe any specific overlaps [35]. Several reasons may account for these differences. Different studies used normal or cancer cell lines. The mode of MYC deregulation (overexpression vs. inducible) and differences between siRNA and shRNA could also account for some differences. Furthermore, MYC regulates multiple cellular processes and its function as well as the impact of its expression depends heavily on the particular cellular context. Finally, it has been proposed that MYC acts as a general amplifier of activated transcription [43, 44], which could explain in part why MYC may be regulating the expression of different genes in different cell types. Thus, while the differences observed between datasets are puzzling, they demonstrate the importance of studying MYC biology within highly controlled and specific contexts that best mimic physiological systems.

In the present study, we have identified 78 genes which down-regulation is synthetic lethal with MYCER activation in MCF10A-MYCER cells. Though our system utilizes a stably expressed fusion MYCER protein to model MYC deregulation, instead of endogenous MYC, previous studies have established that MYCER does not respond to estrogen and faithfully models MYC deregulation [31]. Nevertheless, we still cannot rule out that some of the MYC-SL candidates we identified may associate with cellular complexes not normally associated with MYC. Nevertheless, MYCER was shown to be a valid model for MYC deregulation in a screen that identified the SUMOylation machinery as a MYC-SL pathway using human mammary epithelial cells [29]. Moreover, we confirmed that T47D cells are hypersensitive to FBXW7 knockdown in the absence of experimental modulation of MYC levels; further supporting the idea that MYCER is a valid model for MYC deregulation.

We have validated FBXW7 as a gene which knockdown causes significantly decreased viability for cells with activated MYCER. Though FBXW7 is known to target several oncogenes for degradation, our findings indicate that knockdown of FBXW7 in the presence of MYCER activation causes preferential stabilization of MYCER. Our data suggest that stabilization of MYCER is critical to cause increased accumulation of FBXW7 knockdown cells in S/G2 phase and loss of viability. Of note, we were not able to detect significant levels of DNA damage or apoptosis markers at any given time throughout the course of the competition assays. This may be attributed to the slow and gradual nature of cell death induction in our experiments, mimicking physiological conditions. We observe, at most, 50% decrease in viability of FBXW7 knockdown cells compared to control cells over the course of four weeks. We believe that at any given time point, only a small fraction of cells is expressing markers of DNA damage or apoptosis, which is in contrast to what is seen following acute MYC overexpression. Nevertheless, the possibility of senescence or alternative cell death pathway activation cannot be excluded.

FBXW7 is one of only four MYC-SL candidate genes that were identified from both our screen and Kessler's screen, emphasizing its potential significance as a major MYC-SL pathway [29] (Supplementary Table 1). The two other MYC-SL datasets did not include FBXW7, however, the smaller RNAi libraries utilized in these screens may not have included FBXW7 [30, 35]. Notably, Liu's screen that interrogated the human kinome identified GSK3-β, the kinase responsible for a MYC phosphorylation that is required for recognition by FBXW7 [12, 35]. While it has been very challenging to identify common MYC-SL genes and pathways across available datasets [36], these findings suggest that the FBXW7-dependent MYC degradation pathway may be a *bona fide* MYC-SL pathway shared by multiple cell types.

It is well established that FBXW7 is a crucial component of the SCF-like ubiquitin ligase complex, which targets poly-ubiquitinated proteins for proteasomal degradation. Since many targets of FBXW7 are oncogenes such as MYC, CyclinE, NOTCH1, and c-JUN, FBXW7 is generally considered a tumor suppressor gene. In fact, in human T-cell acute lymphoblastic leukemia (T-ALL), FBXW7 is one of the most frequently mutated genes (approximately 30% of cases [45]) and mouse models with tissue specific knockout of FBXW7 in various settings develop both hematopoietic and solid tumors [46], corroborating the tumor suppressing functions of FBXW7 in these contexts.

We propose that FBXW7 can also act as a tumor maintenance gene in the context of MYC deregulation. For cells expressing MYC at aberrant levels, the presence of FBXW7 enables their continued proliferation and survival. Consistent with our results, increased accumulation of MYC is responsible for the loss of leukemic stemness in leukemia-initiating cells lacking FBXW7 [47-49]. In a T-ALL mouse model, MYC protein levels in leukemia-initiating cells were found to be heavily dependent on FBXW7 activity, demonstrating that MYC is the major contributor to the FBXW7 phenotype [49]. In agreement, our studies suggest that accumulation of active MYC protein is the major contributor to the synthetic lethal phenotype seen upon FBXW7 knockdown and MYCER activation. Though we have not tested other documented targets of FBXW7 such as SREBP, p100, NRF1, NF1, Mcl-1, KLF5, c-Myb, and Aurora A [46], our results strongly suggest that MYCER is the critical target of FBXW7 contributing to the synthetic lethal phenotype in MYCER activated cells.

While FBXW7 mutations are found in several diverse human cancer types such as T-ALL, cholangiocarcinoma, stomach, colon, pancreas, and endometrium, they are rare in other cancers [50]. In fact, according to Akhoondi et al. [18], FBXW7 mutations appear to be infrequent in breast and ovarian cancers. This could be explained in part by our results suggesting that inactivating mutations of FBXW7 may not be able to coexist with MYC overexpression in the breast context. Interestingly, our analysis suggests that MYC and FBXW7 expression are mildly but significantly positively correlated, specifically in the Luminal A-subtype of breast cancers. Notably, this subtype is typically associated with low to normal MYC expression, suggesting that cells retain physiological regulation of MYC abundance [51]. This is in contrast to cells derived from breast cancer types with MYC overexpression, which can be unresponsive to MYC suppression [52]. Breast cancer cells with low MYC expression, such as Luminal A, may be more sensitive to small fluctuations in MYC protein levels which arise from altered FBXW7 expression.

To gain further insight into the relationship between FBXW7 and MYC in breast, further studies are critical. Nevertheless, our results demonstrating the identification of FBXW7 as a synthetic lethal target in the context of MCF10A-MYCER adds insight to the still enigmatic role of the interaction between FBXW7 and MYC in human cancer initiation, progression, and maintenance.

## MATERIALS AND METHODS

### Isolation of MYCER cells

MYCER was introduced retrovirally using pBabe-hygro-MYCER into MCF10A cells and transduced clones were selected by the addition of 100цg/ml Hygromycin B (Roche Holding AG, Basel, Switzerland). Clones were isolated by serial dilution.

### Production of lentivirus and shRNA

Glycerol stocks of lentiviral shRNA constructs (pGIPZ, pTRIPZ) were obtained from Thermo Scientific Open Biosystems (Waltham, Massachusetts, USA) and grown in LB medium with 100цg/ml carbenicillin (Sigma) and 25цg/ml Zeocin (InvivoGen, San Diego, CA, USA). Clones used were FBXW7-7 (V2LHS_202932), FBXW7-8 (V2THS_89328), FBXW7-10 (V2THS_203045), UBE2I-3 (V2LHS_171776), and UBE2I-6 (V3LHS_376933). Plasmids were isolated using the E.Z.N.A. Plasmid Miniprep kit (Omega Bio-Tek, Inc., Norcross, GA, USA) and packaged into lentivirus by transfecting (jetPEI by Polyplus-transfection S.A., Illkirch, France) into 293T cells with pMD.G and pCMVR8.91. Viruses were collected in MCF10A media, filtered, and infected into MCF10A in the presence of 8цg/ml polybrene (Sigma) and spin infected for 1 hr at 1000rpm at RT. After 24 hr of incubation, infected cells were selected by the addition of 2цg/ml puromycin (Sigma).

### Cell culture

MCF10A cells were cultured in DMEM/F12 (Invitrogen, Carlsbad, CA, USA) supplemented with 5% horse serum (Invitrogen), 20ng/ml EGF (Peprotech, Rocky Hill, NJ, USA), 0.5цg/ml hydrocortisone (Sigma-Aldrich Corporation, St. Louis, MO, USA), 100ng/ml cholera toxin (Sigma), 10цg/ml insulin (Sigma), and antibiotics. T47D and 293T cells were cultured in DMEM (Invitrogen) supplemented with 10% FBS (Invitrogen) and antibiotics. All cells were incubated at 37ºC and 5% CO_2_.

### Cell lysis and cellular fractionation

Cells were lysed in RIPA buffer (150mM NaCl, 1% NP40, 0.5% DOC, 0.1% SDS, 50mM Tris pH 8.0), supplemented with protease and phosphatase inhibitors (Sigma), for 15 min on ice. Samples were sonicated (2 × 5 min) and centrifuged for 10 min at 4ºC. Cellular fractionation was performed as published by Mendez and Stillman [38].

### shRNA screen

The pooled shRNA screen was carried out using the Thermo Scientific Open Biosystems GIPZ Lentiviral Human shRNA-mir Library as described in Rodriguez-Barrueco et al [33]. The library was packaged into lentivirus as described above, and MCF10A-MYCER cells were infected at 30% efficiency. Infected cells were selected with 2цg/ml puromycin for two passages and divided into 10 independent populations. Five populations were treated with 200nM 4OHT (every 48 hr) and five were treated with vehicle. After 30 days, genomic DNA of surviving cells was isolated by lysing cells with DNA lysis buffer (1% SDS, 100mM EDTA pH8.0, 50mM Tris-HCl pH8, 100nM NaCl), treating with RNAse A (Qiagen N.V., Venlo, Netherlands), and purifying DNA with phenol chloroform extraction and isopropanol precipitation. Resulting DNA was subjected to PCR to recover the shRNA and associated barcodes, generating a heterogeneous pooled product of approximately 350 bps. The PCR product was gel-extracted and purified, labeled with Cy3 and Cy5 (Roche), and hybridized to customized microarrays (Agilent Technologies, Inc., Santa Clara, CA, USA) harboring complementary probes to the shRNA barcodes. Data analysis was then performed as described in Yu et al [34].

### mRNA isolation and RT-PCR

RNA was isolated using the Nucleospin RNA II kit (Clontech Laboratories, Inc., Mountain View, CA, USA). 1μg of RNA was subjected to reverse transcription using the High Capacity cDNA Reverse Transcription Kit (Applied Biosystems, Life Technologies, Carlsbad, CA, USA). cDNA was used as template for quantitative RT-PCR using ABsolute Blue QPCR SYBR Green Low ROX Mix (Thermo Scientific) and Applied Biosystems 7500 Fast (Life Technologies, Carlsbad, CA, USA).

### Competition assays

250 000 MCF10A-MYCER cells expressing stably integrated shRNA and 250 000 MCF10A-MYCER cells without shRNA were plated together. At the first passage, fluorescent cells were counted by live cell flow cytometry, and replated with either vehicle or 4OHT. At each subsequent passage, remaining percentage of fluorescent cells was analyzed using flow cytometry. All cell sorting was performed with the BD FACSCalibur (BD Biosciences, San Jose, CA, USA).

### Luciferase assay

MCF10A-MYCER cells were plated in 12-well plates. After 24 hr, at 90% confluence, they were transfected with MYC reporter and Renilla plasmid and incubated for 48 hr with or without 4OHT. Using the Dual-Luciferase Reporter Assay System (Promega Corporation, Fitchburg, WI, USA), relative luciferase activity was measured with the GloMax Luminometer (Promega).

### Double thymidine block and indirect immunofluorescence

MCF10A-MYCER cells were plated on sterilized coverslips. After 24 hr, cells were incubated in 2.5mM thymidine for 17 hr. After a 7 hr recovery, the second block was administered at 2.5mM for 14 hr. Vehicle or 4OHT was added 3 hr prior to release. After 1 hr recovery, cells were treated with an 8 min pulse of 33цM BrdU. Immediately, cells were washed and fixed with 4% paraformaldehyde for 15 min at RT, washed, then permeabilized with 0.2% Triton-X in PBS for 5 min and washed. DNA was denatured in 2N HCl for 20 min at RT, following neutralization with 0.1M sodium tetraborate pH8.5. Primary antibody incubation with anti-BrdU (#555647, BD Pharmingen, San Jose, CA, USA) was performed in PBS-0.5%Tween for 30 min at RT, followed by washing, and secondary antibody incubation with goat-anti-mouse-FITC (Jackson ImmunoResearch Laboratories, Inc., West Grove, PA, USA) was performed in PBS-0.5%Tween for 30 min. Cells were washed and stained for 1 min with propidium iodide, airdried, and mounted on slides for confocal microscopy. The Zeiss LSM 510 Meta Inverted (Carl Zeiss AG, Oberkochen, Germany) was used for all confocal imaging.

### Western blotting and quantification

Cell lysates were prepared as described, added to 5X Laemmli buffer and heated at 95ºC for 5 min. Samples were run on Tris-Glycine or NuPAGE Tris-Acetate gels (Invitrogen) and transferred to PVDF membranes at 35V for 2 hr. Membranes were blocked in PBS-0.5%Tween-5%milk for 1 hr and stained with primary antibodies (1:250-1:2000) for 1 hr or overnight, followed by staining with secondary antibodies (1:10,000) conjugated with HRP (Jackson) for 1 hr. After incubation with ECL (Pierce, Thermo Fisher Scientific), blots were exposed to film. ImageQuant 5.2 (Molecular Dynamics) was used for quantification.

### mRNA expression analysis

RNAseq (IlluminaHiSeq) data from TCGA breast invasive carcinoma dataset was downloaded from the cancer browser (TCGA_BRCA_exp_HiSeqV2). Overall, 160 samples with RNAseq data were previously classified as LUMINAL A subtype according to the integrative multiplatform approach described in Nature 2012 (PMID:23000897). Briefly, breast tumors were clustered using a “cluster of clusters” approach based on miRNAs, DNA methylation, copy number, PAM50 mRNA expression, and RPPA expression data. Expression of MYC and FBXW7 in LUMINAL A subtypes were estimated in RSEM normalized count and Pearson correlation coefficient (rho) was calculated using STATA (Stata Statistical Software: Release 12. College Station, TX: StataCorp LP).

### Cell cycle analysis

At the time of harvest, MCF10A-MYCER cells were vortexed in 0.5 ml PBS, and 5 ml of 70% ethanol was added dropwise for fixing at −20ºC overnight. The next day, cells were incubated in 1 ml PBS for 1 hr on ice and centrifuged. To the resulting pellet, RNAse A was added at 1/400, and propidium iodide stock (10mg/ml, Sigma) was added at 1/1000 for flow cytometry. All analysis was performed with the BD FACSCalibur (BD Biosciences).

### Antibodies

The following antibodies were used for immunostaining: MYC (9E10, Santa Cruz Biotechnology, Inc., Dallas, TX, USA), c-Jun (sc-44, Santa Cruz), cleaved Notch1 (4147, Cell Signaling,), cyclin E (sc-481, Santa Cruz Biotech), mTOR (2972, Cell Signaling Technology, Inc., Danvers, MA, USA), βactin (A2228, Sigma), Histone H3 (9715, Cell Signaling), Cdc45 (EPR5759, Epitomics, Burlingame, CA, USA), Phospho-Chk2 (2661, Cell Signaling), Phospho-H2AX (JBW301, EMD Millipore, Billerica, MA, USA), Cleaved Caspase-3 (9664, Cell Signaling), PARP-1 (sc-8007, Santa Cruz), PUMA (sc-28226, Santa Cruz).

### Statistics

All statistical values represented in figures were generated through appropriate t-tests (paired, unpaired, one-sample or two-sample) using Prism 5 (GraphPad Software, La Jolla, CA, USA).

## SUPPLEMENTARY FIGURES AND TABLES


