# Supplementary material for: MYC is a critical target of FBXW7
Source: Oncotarget. 2014 Dec 26;6(5):3292–305. doi: 10.18632/oncotarget.3203 (PMC4413654; doi:10.18632/oncotarget.3203)
Supplement: Supplementary file 1 [file oncotarget-06-3292-s001.pdf]

MYC is a critical target of FBXW7

Supplementary Material

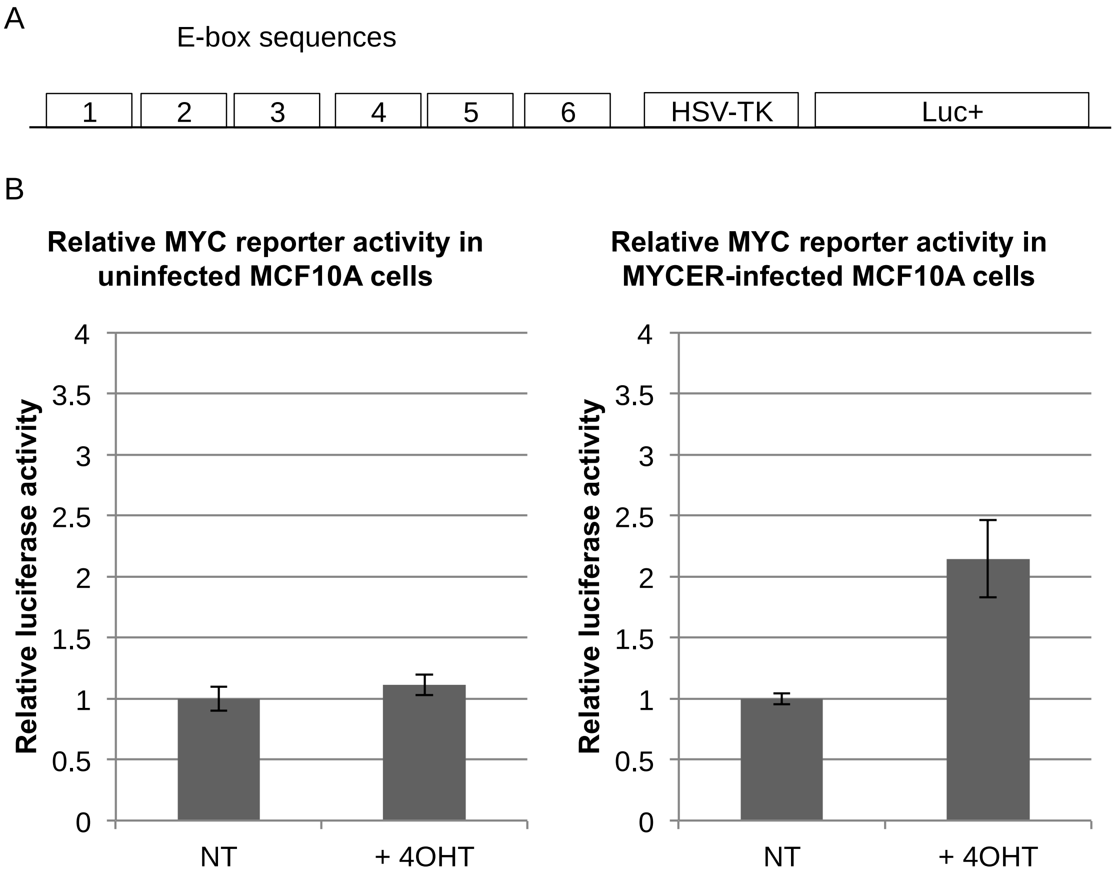

Supplementary Figure 1: MYCER protein expressed from the transgene can enhance transcriptional activity. (A) A reporter plasmid containing 6 E-box sequences (CACGTG) followed by a luciferase reporter was transfected into uninfected parental MCF10A or MCF10A-MYCER cells and luminescence was measured 48 hr after transfection. (B) Luciferase activity from the reporter was normalized to Renilla luciferase activity. Each experiment was done in triplicate and graphs show the average of 3 biological replicates. Error bars represent the SEM.

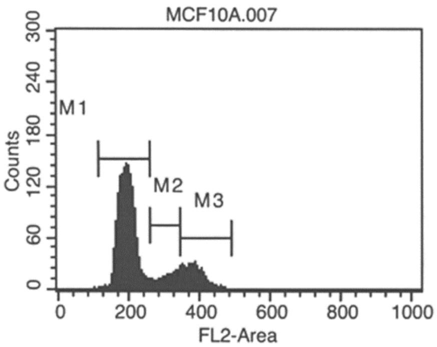

Asynchronous cells

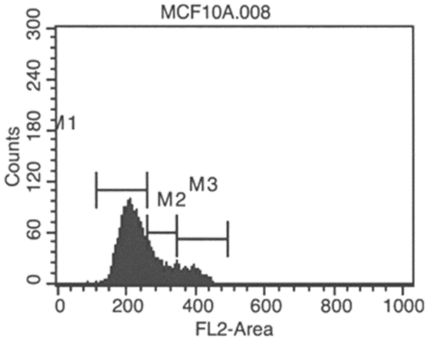

After double thymidine block

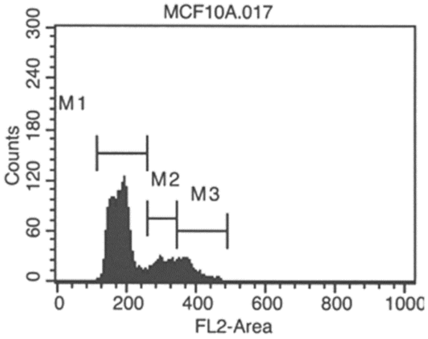

24h post release from DT block

Supplementary Figure 2: MCF10A-MYCER cells arrest at the G1/S transition following double thymidine block. MCF10A-MYCER cells were subjected to two rounds of 2.5mM thymidine treatment, then fixed and stained with propidium iodide for flow cytometric analysis. Compared to untreated asynchronous cells, thymidine treated cells showed accumulation of cells in M1+M2 (<2N DNA content). 24h after release, the cell cycle distribution returns to normal.

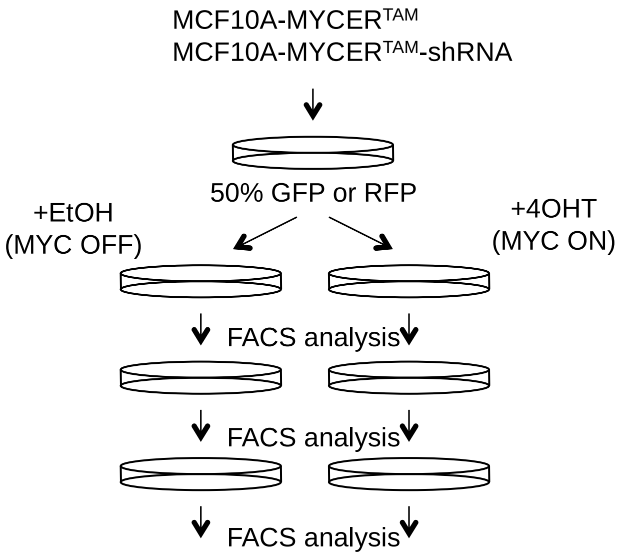

Supplementary Figure 3: Schematic of fluorescence-based competition assay. Uninfected MCF10A-MYCER cells which express no fluorescent proteins are plated on the same plate with MCF10A-MYCER cells infected with a single shRNA clone of choice co-expressing GFP or RFP. At day 1, cells from the plate are subjected to live cell flow cytometry to verify ~50% fluorescence. Subsequently, the cells are split into control or 4OHT treated groups, and passaged every 48-72 hr. At each passage or desired timepoint, remaining fluorescence on the plate is measured by flow cytometry for the duration of the experiment.

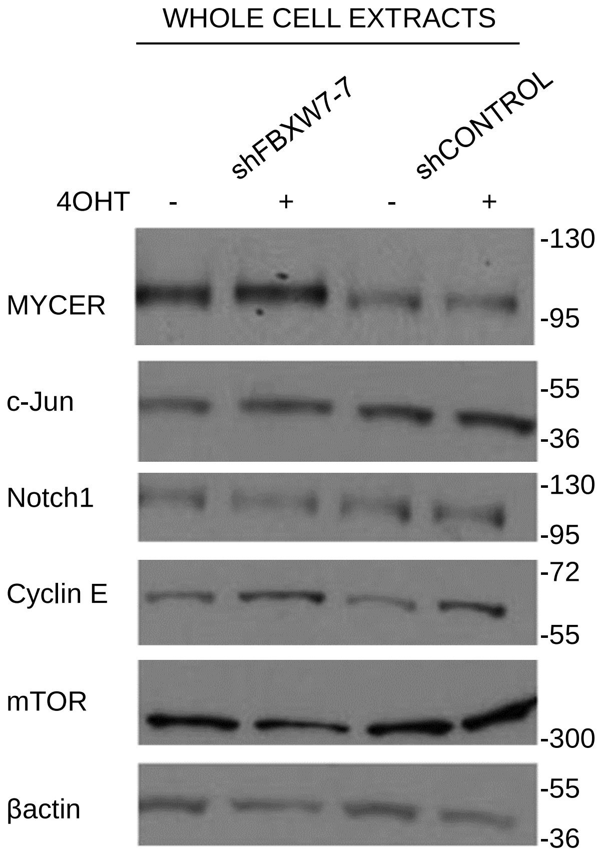

Supplementary Figure 4: FBXW7 knockdown specifically leads to stabilization of MYCER. MCF10A-MYCER cells expressing either control or FBXW7 shRNA were cultured in the presence or absence of 4OHT for 4 weeks. Whole cell lysates (80µg total protein/lane) were run on SDS-PAGE and subjected to Western blot for analysis of major FBXW7 targets. Figure shows a representative image of replicated experiments.

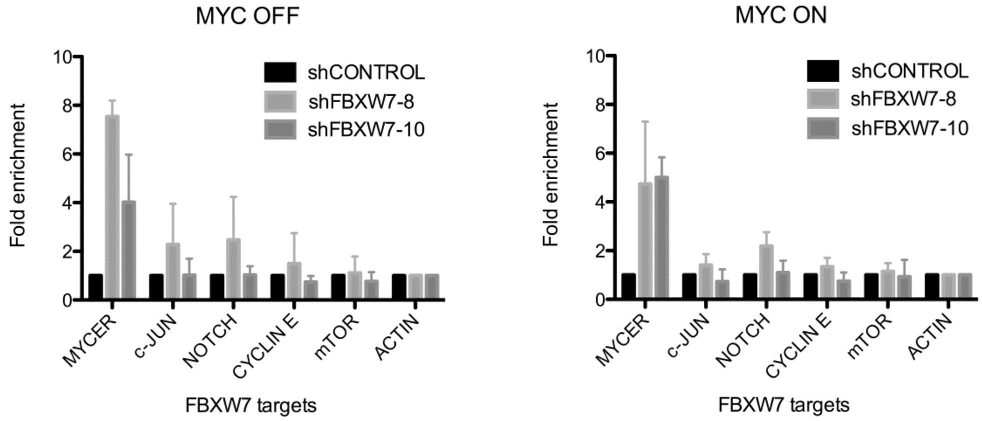

Supplementary Figure 5: FBXW7 knockdown leads to preferential stabilization of MYCER. MCF10A-MYCER cells stably expressing Dox-inducible shRNA clones of FBXW7 were treated with or without 4OHT (MYC OFF or ON) for 4 weeks, then cell lysates were analyzed by SDS-PAGE/Western blot. Bands were quantified using ImageQuant v5.2 (Molecular Dynamics) and normalized to loading controls. Figure shows averages from 3 independent biological replicates.

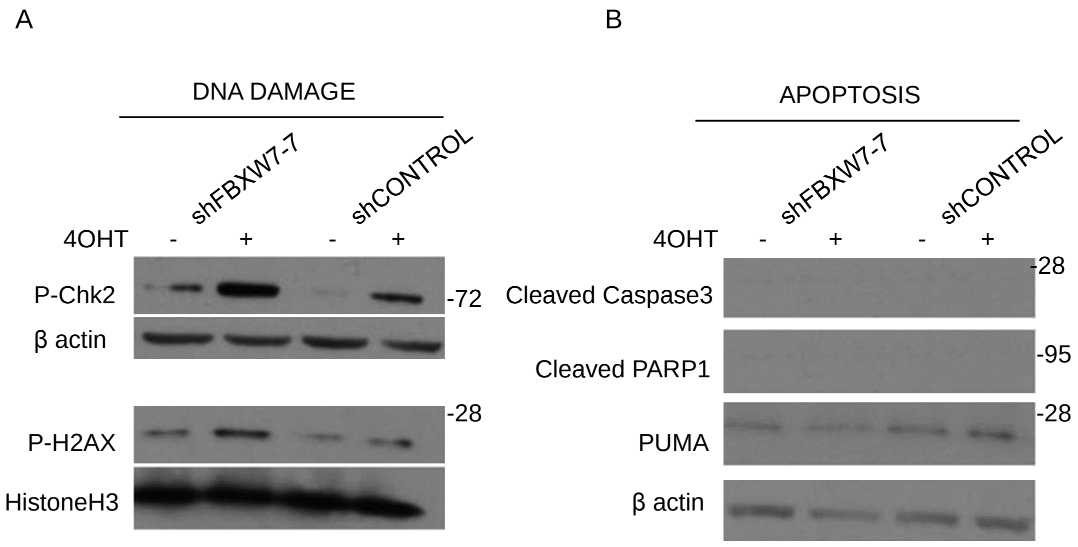

Supplementary Figure 6: Long term FBXW7 knockdown and MYCER activation do not result in significant levels of DNA damage or apoptosis. (A) Control or FBXW7 knockdown cells were treated with or without 4OHT for 4 weeks and probed for the presence of phosphorylated Chk2 (in whole cell lysate) or H2AX (in chromatin-bound samples after fractionation) as markers of checkpoint activation/DNA damage. shFBXW7 cells did not show a significant accumulation of either species after long term treatment with 4OHT. (B) Similarly treated cells were probed for the presence of apoptosis markers in whole cell lysates. However, we failed to detect any significant levels of cleaved caspase-3, PARP1, or PUMA in the surviving cells after 4 weeks of treatment. Shown above are representative images of 3 independent replicates.

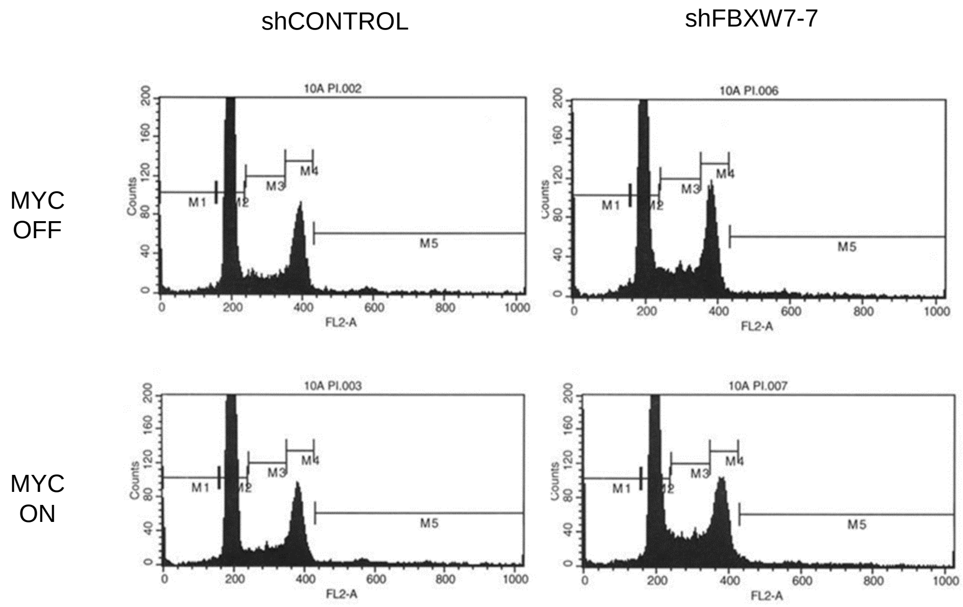

Supplementary Figure 7: FBXW7 knockdown with MYCER activation causes synergistic accumulation of cells in S and G2/M phase. MCF10A-MYCER cells stably expressing control or FBXW7 shRNA were cultured for 4 weeks in the presence or absence of 4OHT (MYC OFF or ON). Cells were fixed and stained with propidium iodide for flow cytometric analysis. Cells accumulate in the M3+M4 region (<1N DNA content) only in the FBXW7 knockdown and MYC ON condition. Shown is a representative image of 3 independent replicates.

Supplementary Table 1: List of top 78 synthetic lethal targets from MCF10A-MYCER screen. Results were filtered according to fold change (Log2 FC<-1) of signal abundance between MYC ON and MYC OFF, and individually associated p-values (p<0.05). Only annotated target genes are shown. Candidates are ranked in order of most depleted in the MYC ON population compared to MYC OFF (Log2 FC value).

Rank	Name	p-value	Log2 FC	Acc. Number	shRNAi ID
1	ARL5B	0.0500	-2.7089	NM_178815	v2HS_118631
2	C2orf44	0.0100	-2.5672	NM_025203	v2HS_137381
3	TTBK1	0.0300	-2.4316	AB058758	v2HS_238991
4	PSG6	0.0400	-2.3989	NM_002782	v2HS_170657
5	NAT9	0.0500	-2.3896	NM_015654	v2HS_263964
6	TRPM6	0.0300	-2.1357	NM_017662	v2HS_155076
7	MFN2	0.0100	-2.0827	NM_014874	v2HS_95806
8	C18orf54	0.0100	-1.8569	NM_173529	v2HS_160761
9	DONSON	0.0300	-1.8499	NM_017613	v2HS_154816
10	ZNF614	0.0400	-1.8499	NM_025040	v2HS_235983
11	H2AFV	0.0100	-1.8450	NM_138635	v2HS_200741
12	NUPL1	0.0500	-1.8381	NM_014778	v2HS_233311
13	ZNF691	0.0100	-1.7257	NM_015911	v2HS_236174
14	PSMD2	0.0500	-1.7213	NM_002808	v2HS_219275
15	CDC14B	0.0400	-1.7209	NM_033331	v2HS_203260
16	UGT1A1	0.0300	-1.6796	NM_000463	v2HS_93183
17	SOX15	0.0500	-1.6110	NM_006942	v2HS_229072
18	NUPL2	0.0500	-1.6086	NM_007342	v2HS_86275
19	ANKRD27	0.0500	-1.6068	NM_032139	v2HS_117932
20	EPHA7	0.0100	-1.5984	NM_004440	v2HS_17738
21	ST8SIA4	0.0500	-1.5098	NM_005668	v2HS_238967
22	SLC44A2	0.0500	-1.4952	NM_020428	v2HS_72471
23	SH3BP4	0.0300	-1.4934	NM_014521	v2HS_86832
24	KIAA0509	0.0300	-1.4922	AB007978	v2HS_259524
25	DMKN	0.0500	-1.4804	NM_033317	v2HS_160312
26	STK17A	0.0100	-1.4726	NM_004760	v2HS_36021
27	PHF21A	0.0100	-1.4564	NM_016621	v2HS_135309
28	EYA2	0.0200	-1.4531	NM_005244	v2HS_43093
29	C6orf184	0.0100	-1.4413	XM_168053	v2HS_121031
30	ZFYVE9	0.0100	-1.4407	NM_004799	v2HS_35331
31	LRCH3	0.0400	-1.4395	NM_032773	v2HS_222832
32	NR2E1	0.0100	-1.4308	NM_003269	v2HS_261941
33	TOP3B	0.0100	-1.4280	NM_003935	v2HS_47174
34	WDSOF1	0.0300	-1.4122	NM_015420	v2HS_229697
35	CLDN5	0.0500	-1.3596	NM_003277	v2HS_171412
36	CMTM2	0.0400	-1.3528	NM_144673	v2HS_17530
37	IRAK1	0.0300	-1.3447	NM_001569	v2HS_132369

38	APOBEC3A	0.0300	-1.3348	NM_145699	v2HS_286556
39	ERGIC1	0.0300	-1.3258	NM_020462	v2HS_71567
40	PARP15	0.0100	-1.3206	NM_152615	v2HS_44475
41	RAE1	0.0500	-1.2995	NM_003610	v2HS_27966
42	LEPREL1	0.0500	-1.2897	NM_018192	v2HS_156224
43	ZNF132	0.0300	-1.2802	NM_003433	v2HS_172208
44	SLC7A1	0.0500	-1.2710	NM_003045	v2HS_153014
45	LAMA2	0.0200	-1.2692	NM_000426	v2HS_92966
46	LOC344065	0.0400	-1.2685	XM_292895	v2HS_143532
47	ATP1B1P1	0.0500	-1.2641	NG_001081	v2HS_169198
48	C1orf182	0.0500	-1.2536	NM_144627	v2HS_18680
49	KRIT1	0.0400	-1.2463	NM_004912	v2HS_62790
50	ASAH3	0.0400	-1.2436	NM_133492	v2HS_99964
51	OLFR89	0.0300	-1.2192	AJ132194	v2HS_18007
52	CCDC25	0.0300	-1.1758	NM_018246	v2HS_156496
53	TOP3A	0.0100	-1.1740	NM_004618	v2HS_42272
54	MATK	0.0200	-1.1567	NM_002378	v2HS_203423
55	ST14	0.0100	-1.1516	NM_021978	v2HS_228778
56	KIAA0574	0.0300	-1.1513	AB011146	v2HS_130193
57	PERP	0.0200	-1.1513	NM_022121	v2HS_216968
58	FBXW7	0.0200	-1.1469	AY033553	v2HS_89326
59	APOA2	0.0100	-1.1369	NM_001643	v2HS_132594
60	C5	0.0400	-1.1342	NM_001735	v2HS_150150
61	SLC36A4	0.0500	-1.1260	NM_152313	v2HS_25433
62	PPP3CC	0.0300	-1.1253	NM_005605	v2HS_57300
63	TRIM26	0.0500	-1.1229	NM_003449	v2HS_172289
64	ELL	0.0500	-1.1181	NM_006532	v2HS_198413
65	PRMT8	0.0100	-1.1163	NM_019854	v2HS_34353
66	PRSS21	0.0500	-1.1146	NM_006799	v2HS_83969
67	UBE2I	0.0200	-1.0971	NM_003345	v2HS_171781
68	QRSL1	0.0400	-1.0967	XM_301163	v2HS_49076
69	C11orf48	0.0400	-1.0951	NM_024099	v2HS_116419
70	EPYC	0.0400	-1.0697	NM_004950	v2HS_61963
71	TNFAIP2	0.0100	-1.0598	NM_006291	v2HS_56492
72	PAQR3	0.0300	-1.0499	NM_177453	v2HS_211154
73	PREB	0.0500	-1.0457	NM_013388	v2HS_64102
74	CEND1	0.0300	-1.0194	NM_016564	v2HS_135015
75	BBOX1	0.0300	-1.0107	NM_003986	v2HS_246341
76	CDKL5	0.0500	-1.0097	NM_003159	v2HS_262311
77	CMTM7	0.0100	-1.0049	NM_138410	v2HS_70332
78	GCN1L1	0.0400	-1.0020	XM_045792	v2HS_68924
